# Family-Based Genome-Wide Association Study of Autism Spectrum Disorder in Middle Eastern Families

**DOI:** 10.3390/genes12050761

**Published:** 2021-05-18

**Authors:** Yasser Al-Sarraj, Eman Al-Dous, Rowaida Z. Taha, Dina Ahram, Fouad Alshaban, Mohammed Tolfat, Hatem El-Shanti, Omar M.E. Albagha

**Affiliations:** 1 College of Health and Life Sciences, Hamad Bin Khalifa University, Doha 34110, Qatar; yalsarraj@qf.org.qa (Y.A.-S.); eymaldous@hbku.edu.qa (E.A.-D.); 2 Qatar Biomedical Research Institute (QBRI), Hamad Bin Khalifa University, Doha 34110, Qatar; rtaha@hbku.edu.qa (R.Z.T.); da2747@cumc.columbia.edu (D.A.); falshaban@hbku.edu.qa (F.A.); hatem-el-shanti@uiowa.edu (H.E.-S.); 3 Division of Nephrology, Columbia University Medical Center, New York, NY 10032, USA; 4 The Shafallah Center for Children with Special Needs, Doha 33123, Qatar; Mohamed.Tolefat@shafallah.org.qa; 5 Department of Pediatrics, Carver College of Medicine, University of Iowa, Iowa City, IA 52242, USA; 6 Centre for Genomic and Experimental Medicine, Institute of Genetics and Molecular Medicine, University of Edinburgh, Edinburgh EH4 2XU, UK

**Keywords:** autism spectrum disorder, neuropsychiatric disorders, genetic, genome-wide association, family

## Abstract

Autism spectrum disorder (ASD) is a neurodevelopmental disease characterized by abnormalities in language and social communication with substantial clinical heterogeneity. Genetic factors play an important role in ASD with heritability estimated between 70% to 80%. Genome-wide association studies (GWAS) have identified multiple loci associated with ASD. However, most studies were performed on European populations and little is known about the genetic architecture of ASD in Middle Eastern populations. Here, we report the first GWAS of ASD in the Middle eastern population of Qatar. We analyzed 171 families with ASD, using linear mixed models adjusting for relatedness and other confounders. Results showed that common single nucleotide polymorphisms (SNP) in seven loci are associated with ASD (*p* < 1 × 10^−5^). Although the identified loci did not reach genome-wide significance, many of the top associated SNPs are located within or near genes that have been implicated in ASD or related neurodevelopmental disorders. These include *GORASP2, GABBR2, ANKS6, THSD4, ERCC6L*, *ARHGEF6*, and *HDAC8*. Additionally, three of the top associated SNPs were significantly associated with gene expression. We also found evidence of association signals in two previously reported ASD-susceptibility loci (rs10099100 and rs4299400). Our results warrant further functional studies and replication to provide further insights into the genetic architecture of ASD.

## 1. Introduction

Autism spectrum disorder (ASD) is characterized by aberrations in social interaction and communication that are associated with repetitive behaviors and interests, with substantial clinical heterogeneity [1]. The worldwide prevalence of ASD in children has been estimated to be approximately 1% and is about four times more common in males than females [2]. Recently, a cross-sectional survey of the Middle Eastern Qatari population between 2015 and 2018 revealed an ASD prevalence of 1.14% (95% CI: 0.89–1.46) among children aged between 6 and 11 years old [3]. There are neuropathological changes among subjects with ASD that usually occur at the early stages of brain development and influence the functional connectivity and synaptic plasticity, as well as neurotransmission [4,5,6,7]. Genetic factors play an important role in the etiology of ASD; twin studies have shown that the heritability of ASD ranges from 70% to 80% [8,9]. However, ASD is highly heterogenous in terms of clinical presentation, as well as the underlying genetic architecture. Studies using massively parallel sequencing have identified many rare de novo pathogenic variants in ASD patients [10,11,12,13]. Additionally, copy number variants (CNVs), including large deletions and duplications, have been detected across the genome in patients with ASD [14,15,16,17]. However, these variants are rare and explain a small proportion of ASD heritability, suggesting that the majority of the genetic risk of ASD could be attributed to common genetic variations [18]. Although earlier genome-wide association studies (GWAS) identified common variants and susceptibility loci, none reached genome-wide significance and a few showed consistent association [19,20,21,22]. Nonetheless, a recent large scale GWAS of about 18,000 ASD cases and 28,000 controls identified five ASD-susceptibility loci with genome-wide significance [23]. Another large-scale meta-analysis of GWAS in ASD identified a susceptibility locus on chromosome 10q24.32 [24]. However, most GWAS studies of ASD were performed on European populations [19,20,21,22,23,24,25,26], and a few on Chinese and Korean [27,28,29], but none in Middle Eastern populations.

Here, we report the first ASD-related GWAS in the Middle Eastern population of Qatar using a family-based approach. We identified multiple ASD susceptibility loci with suggestive evidence of association with ASD, as some were located within or near genes previously implicated in ASD or related neurodevelopmental disorders. We then investigated the functional relevance of the identified loci and showed that three loci were expression quantitative trait loci (eQTL). Additionally, we found evidence of replication signals in our data for two previously reported loci.

## 2. Materials and Methods

### 2.1. Study Subjects

The study cohort is made of 171 nuclear families (trios), each identified through a proband diagnosed with ASD by either an ADI-R (Autism Diagnostic Interview; revised) [30] or an ADOS (Autism Diagnostic Observation Schedule) [31]. Four families had additional affected siblings to the proband and forty-four families were missing one parent (incomplete trio). A signed informed consent was obtained from the participants or their legally authorized representatives. The probands had a clinical work-up that included an IQ test and other tests to identify associated comorbid conditions. All affected children were examined by a clinical geneticist (H E-S) to exclude any dysmorphic features or congenital anomalies. Due to the nature of the ascertainment source (The Shafallah Center for Children with Special Needs), all probands had associated intellectual impairment, and about 25% had epilepsy. The research project was approved by the Qatar Biomedical Research Institute’s Institutional Review Board (IRB).

### 2.2. Genotyping

DNA was extracted from whole peripheral blood using the Gentra Puregene kit (Qiagen Sciences, Germantown, MD, USA) by following the manufacturer’s guidelines. Genomic DNA quality and quantity were assessed using NanoDrop Spectrophotometer (ThermoFisher Scientific, UK). SNP genotyping was performed using Illumina Infinium Bead Chip Human1M-Duov3 (345 samples) or HumanOmniExpress-12v1-1 (150 samples) (Illumina, San Diego, CA, USA) as per manufacturer’s protocol. The genotyping data and allele calling were conducted using GenomeStudio 2011.1 from Illumina.

### 2.3. Genome-Wide Association Analysis

Standardized quality control measures were applied to filter out low quality data from both SNP and sample levels [32] using PLINK-1.9 [33]. Genotypic data from the two genotyping arrays were merged and the analysis was focused on SNPs that were common to the two arrays (*n* = 545,130). We excluded SNPs with missing call rates > 1% (*n* = 15,415), Hardy–Weinberg equilibrium test *p*-value < 1 × 10^–6^ (*n* = 61), and minor allele frequency (MAF) < 1% (*n* = 13,334), leaving a total of 516,320 SNPs passing quality filters. Furthermore, we removed individuals with missing genotype rate > 5% (*n* = 1) and those with excess heterozygosity (*n* = 1). We then conducted principal components analysis (PCA) using PLINK-1.9 [33] on a pruned set of independent (r^2^ < 0.05) common (MAF > 1%) SNPs (*n* = 58,052) to define the population background and exclude population outliers; and excluded six samples because they were population outliers as defined by their PC1 or PC2 being more than 4 standard deviations away from sample mean values. The final set of 487 participants were included for downstream association analysis. A genomic kinship matrix of all individuals was calculated using the GenABEL package (v1.8.0) [34] implemented in R.3.4.3 [35] to correct for relatedness [34,36]. Association testing was performed using the linear mixed-effect model (LMM) by including gender and the first four population principal components as covariates using the GenABEL package [34], as described previously [37,38,39]. For the X chromosome, loci were coded as (0, 2) for males and (0, 1, 2) for females; the association analysis was performed separately in males and females, and association results were combined using meta-analysis as implemented in the GenABEL package. The pseudoautosomal regions of the X chromosome were not included in the analysis. Furthermore, to assess the relevance of the identified loci to ASD and related phenotypes, we used FUMA [40], PhenoScanner-v2 [41], GWAS catalog [42], and PubMed literature searches. LocusZoom was used to generate regional association plots for the identified loci [43]. The threshold for genome-wide significance was *p* < 5 × 10^−8^, and the threshold for suggestive evidence of association was *p* < 1 × 10^−5^. We then assessed loci reported in previous GWAS of ASD for replication in our dataset. First, we assessed SNPs with robust genome-wide significant association with ASD from previous studies (*p* < 5 × 10^−8^) [23], [24]. For SNPs that were not genotyped in our dataset, we searched for close proxies (within 100 kb) in linkage disequilibrium (LD) with the lead SNP based on LD data of European population form 1000 Genomes. Proxies were then assessed for evidence of replication in our dataset. We also assessed SNPs associated with ASD (*p* < 1 × 10^−5^) from the GWAS catalog [42] for replication in our dataset using “autism” or “autism spectrum disorder” as phenotypes. The binomial sign test was performed using GraphPad software (GraphPad, San Diego, CA, USA).

### 2.4. Polygenic Risk Score Analysis

Polygenic risk scores (PRS) were calculated using the “score” function in PLINK and were weighted by the estimated effect size of risk alleles. We assessed the performance of European-derived PRS in our cohort by calculating PRS based on loci reported by Grove et al. [23] with *p* < 1 × 10^−6^.

### 2.5. Expression Quantitative Trait Locus (eQTL) Analysis

The effect of associated SNPs on gene expression (eQTL) was assessed using the Genotype-Tissue Expression (GTEx) online database (https://gtexportal.org/home/, accessed on 14 April 2021). The SNP “rs” identifier was used as a search term and eQTL plots were generated for the tissue with the highest statistical significance.

## 3. Results

### 3.1. Characterstics of Study Subjects

**Figure 1:**
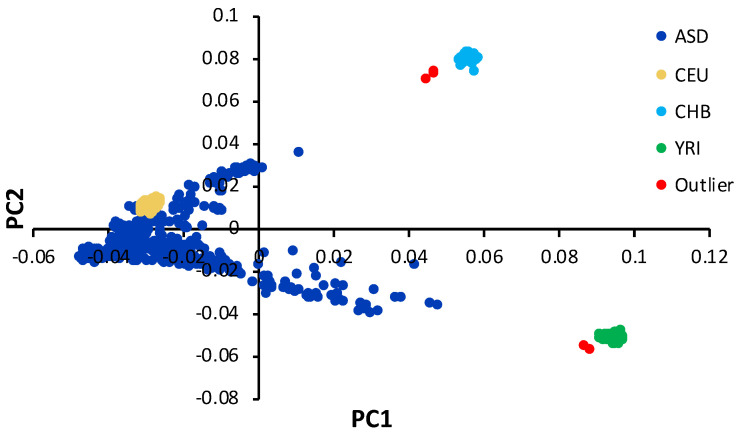
Population background. The first population principal component (PC1) is drawn against the second component (PC2) for study subjects (blue) as well as the three HapMap reference samples including European (CEU), East Asian (CHB) and African (YRI) populations. Population outliers were defined as samples deviating more than 4 standard deviation units from the mean population cluster and are shown in red color.

The majority of study subjects were ASD simplex families with one child with ASD and the parents. Four families had more than one affected child. The average age (± standard deviation) of ASD patients was 8.4 ± 4.3 years. The ratio of male to female children with ASD was 4.6:1. The average age of the parents was 42.1 ± 8.2 years. Supplementary Table S1 shows the family structure of the study subjects. The principal component analysis showed that the majority of study subjects was located within one population cluster. We identified six subjects as population outliers, which were removed before the association analysis (Figure 1).

### 3.2. Genome-Wide Association Analysis of ASD

**Figure 2:**
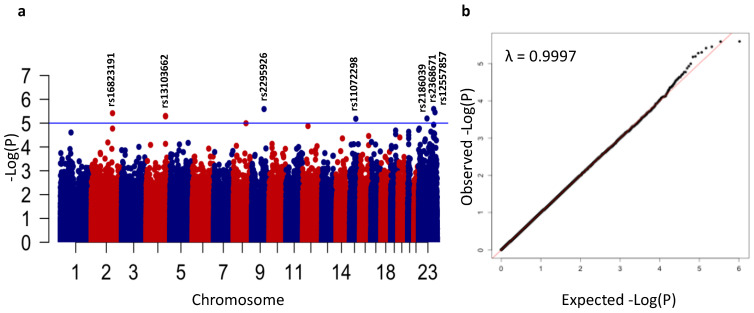
Manhattan and quantile-quantile plots. (**a**) Manhattan plot of association test results showing the chromosomal position of 516,320 analyzed SNPs plotted against −log (*p*). The blue horizontal line represents the threshold for suggestive evidence of association (*p* < 1 × 10^−5^). (**b**) Quantile-quantile plot showing the expected versus observed −log (*p*) values. The genomic inflation factor (λ) is shown at the top left corner.

We used the linear mixed model to investigate the association between SNP genotypes and ASD. We included gender and the first four population PCs in the model to account for any subpopulation stratification, and the genomic kinship matrix to account for relatedness. The association testing results are summarized in the Manhattan plot (Figure 2a), which shows the top signals associated with ASD. The association testing results showed no evidence of inflation (lambda = 0.9997), as shown in Figure 2b.

Seven loci showed suggestive evidence of association (*p* < 1 × 10^−5^) with ASD (Table 1). The first locus is on chromosome 2q31.1 (rs16823191, *p* = 3.8 × 10^−6^). The associated region spans about 450 kb and contains two genes: *TLK1* and *GORASP2* (Supplementary Figure S1a). Interestingly, variants within *GORASP2* are associated with cognitive performance in the GWAS catalog and PhenoScanner [44]. The second locus is located on chromosome 4q32.1 and tagged by rs13103662 (*p* = 4.98 × 10^−6^). The associated region spans about 300 kb, and is bound by two strong recombination hotspots, but does not contain mapped genes (Supplementary Figure S1b). The third locus is located on chromosome 9q22.33 and tagged by rs2295926; this locus shows suggestive evidence of association with ASD (*p* = 2.57 × 10^−6^; Supplementary Figure S2a). This region harbors three protein coding genes: *GABBR2*, *ANKS6*, and *GALNT12*. The fourth locus is on chromosome 15q23 with rs11072298 (*p* = 6.58 × 10^−6^), located within *THSD4* (Supplementary Figure S2b), in which a deleterious de novo mutation (p.P839L) is reported in ASD [11].

The three remaining loci are located on chromosome X. The first is tagged by rs2368671 (*p* = 6.38 × 10^−6^) on Xq13.1 and located within *CITED1* (Supplementary Figure S3a), but this region contains other protein coding genes such as *PIN4*, *ERCC6L*, *RPS4X*, and *HDAC8*. The second is on Xq25 tagged by rs2186039 (*p* = 2.55 × 10^−6^), which is located close to *DCAF12L2* (Supplementary Figure S3b). *DCAF12L2* is located in a region with several reported deletions and duplications in patients with intellectual disability (ID), global developmental delay (GDD), and delayed speech and language development, as well as seizures [45]. The third is on Xq26.3, and spans about 350 kb (Supplementary Figure S4). The observed signal is tagged by rs12557857 (*p* = 3.52 × 10^−6^), and located within *ARHGEF6*, a gene implicated in syndromic and non-syndromic X-linked intellectual disability [46,47].

**Figure 3:**
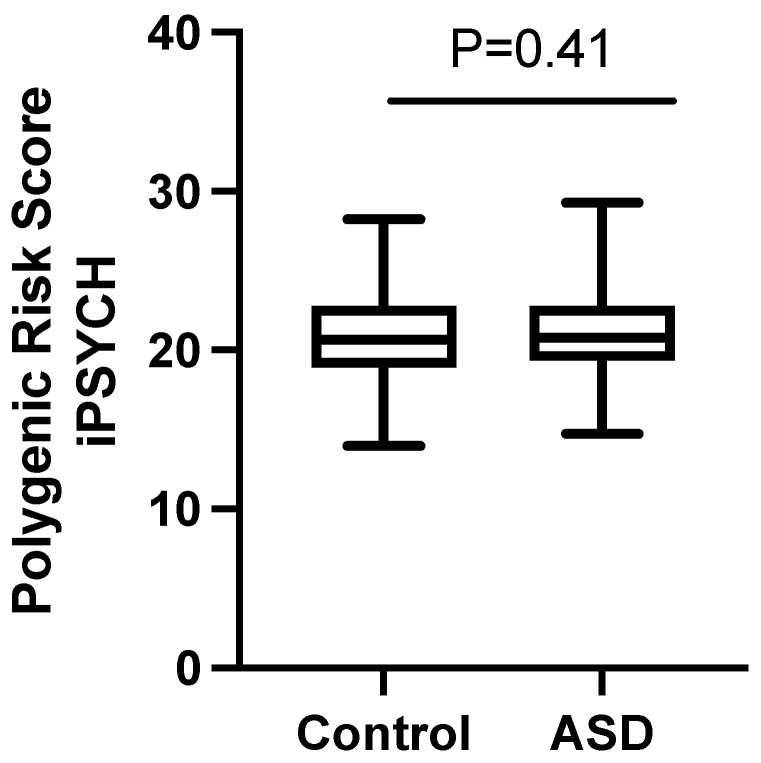
Analysis of polygenic risk scores (PRS). Assessment of PRS defined by SNPs with *p* < 1 × 10^−6^ reported by Grove el al. (iPSYCH) [23] in our cohort. Values are presented as box and whiskers plots showing the interquartile range (boxes), median (horizontal line), the minimum and maximum values (whiskers).

We then assessed the predictive performance of PRS derived from European populations in our cohort by calculating PRS based on loci reported by Grove et al. [23] with *p* < 1 × 10^−6^. Of the 466 SNPs reported by Grove et al., 24 were genotyped in our dataset and were used to calculate PRS. Results showed that European-derived PRS were not significantly associated with ASD in our cohort (OR = 1.03, 95% CI 0.96–1.11; *p* = 0.41), although subject with ASD had slightly higher PRS (21.0) compared to controls (20.8), but this was not statistically significant (*p* = 0.41; Figure 3).

**Figure 4:**
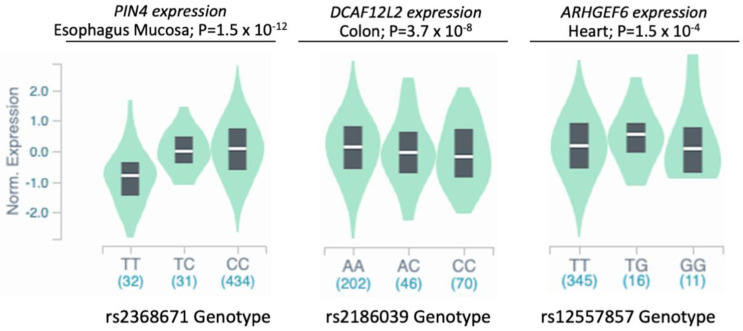
Gene expression in relation to genotypes of ASD-associated SNPs. Bean plots of normalized gene expression in relation to genotypes showing the median (white line) and interquartile range (black box). Data and plots were adapted from the GTEx database [48].

Since none of the suggestively associated SNPs are coding variants, we investigated their functional significance as eQTL using the Genotype-Tissue Expression (GTEx) database. We found that three of the seven top associated SNPs are significant eQTL in multiple tissues (Supplementary Figures S5–S7). eQTL plots from the tissue with highest statistical significance are shown in Figure 4. The risk allele “T” of rs2368671 is associated with reduced *PIN4* gene expression in multiple tissues (Supplementary Figure S5). Similarly, the rs2186039 allele “C” conferring risk of ASD is associated with reduced *DCAF12L2* gene expression (Figure 4 and Supplementary Figure S6) and rs12557857 genotypes are associated with *ARHGEF6* gene expression (Figure 4 and Supplementary Figure S7).

### 3.3. Replication of Loci Reported in Previous GWAS of ASD

We investigated SNPs previously reported in GWAS of ASD in our sample, focusing on variants with genome-wide significant association first. Previous large scale GWAS studies identified six SNPs with robust genome-wide significant association with ASD (*p* < 5 × 10^−8^) [23,24]. These include: rs201910565, rs111931861, rs10099100, rs1409313, rs71190156, and rs910805. Only one SNP (rs910805) was genotyped in our dataset, but this showed no significant association with ASD (*p* > 0.05). However, we found evidence of association in our dataset (*p* < 0.05) for multiple SNPs located close to, and in linkage disequilibrium (LD) with, two previously reported SNPs. For example, we found five SNPs in our dataset that were located within about 50 kb of rs10099100, showed evidence of association with ASD (*p* < 0.05), and were in LD with rs10099100 (Supplementary Table S2). Additionally, an SNP (rs4299400) in our dataset located within 85 kb of rs71190156 showed evidence of association with ASD (*p* = 0.03), and was in LD with rs71190156 (D’ = 0.55). We then assessed SNPs associated with ASD (*p* < 1 × 10^−5^; *n* = 66) from the GWAS catalog [42] for replication in our dataset. Of the 66 SNPs from the GWAS catalog, 29 were genotyped in our dataset, but none showed significant association with ASD (*p* > 0.05; Supplementary Table S3). Comparison of the direction of effect was possible for 12 SNPs and showed a concordance rate of 58.3% (sign test *p*-value = 0.39).

## 4. Discussion

We performed a family-based, genome-wide association analysis of ASD using a cohort from the Middle East and identified seven loci with suggestive evidence for association with autism. The locus on 2q31.1 harbors two genes: *TLK1* and *GORASP2*. *TLK1* belongs to a well-conserved Tousled-like kinases (TLKs) that are involved in various cellular functions including DNA replication and repair, chromatin structure, and regulation of cell cycle. Interestingly, SNPs in *TLK1* have been associated with mathematical ability in GWAS studies [49], whereas pathogenic mutations in *TLK2*, a paralog of *TLK1,* have been reported in patients with ASD, intellectual disability (ID), schizophrenia, and microcephaly [50,51,52,53]. *GORASP2* encodes a member of the Golgi reassembly stacking protein that plays a role in Golgi ribbon formation. A de novo missense mutation in *GORASP2* has been reported in an ASD patient [54], and SNPs within this gene have been associated with cognitive performance in GWAS studies [44]. Furthermore, the 2q31.1 region has been linked to ASD and related pervasive developmental disorders in genome-wide linkage studies [55,56].

The locus on 9q22.33 harbors three genes, *GABBR2*, *ANKS6*, and *GALNT12*. *GABBR2* encodes gamma-aminobutyric acid type B receptor subunit 2, a protein that belongs to the G-protein coupled receptor and GABA-B receptor superfamily. This gene has been implicated in many neurodevelopmental and neuropsychiatric disorders [57,58]. In addition, several pathogenic variants in *GABBR2* have been reported in patients with early infantile epileptic encephalopathy [59], Rett syndrome-like disorders [60,61] and ASD [62]. On the other hand, *ANKS6* encodes a protein that contains multiple ankyrin repeats with possible role in renal and cardiovascular development. Studies have reported on two de novo variants in individuals with ASD from the Simons Simplex Collection [63]; one was intronic [64], and the other was a deleterious missense variant (p.R467Q) [11].

*THSD4* is the only gene located within the 15q23 locus and has been associated, along with a combined set of genes, with “social skills” quantitative trait association analyses conducted in twins [65]. Additionally, a missense de novo variant (p.P839L) in *THSD4* has been reported in an ASD patient from the Simons Simplex Collection [63].

Two genes located within the locus on Xq13.1 have relevance to ASD. Missense de novo variants in *ERCC6L* have been reported in patients with ASD (p.Q932H) [54] and developmental disorders (p.M358T) [66]. The other is *HDAC8,* which is associated with Cornelia de Lange syndrome (CDLS5), a developmental disorder characterized by short stature and intellectual disability with variable clinical presentations [67]. Multiple missense de novo and pathogenic variants have been reported in *HDAC8* in patients with developmental disorders including those with intellectual disability [66]. There is a consensus among researchers that 20–49% of individuals with *HDAC8* pathogenic variants present with ASD [68]. A study reported a stop gain de novo pathogenic variant (p.Y174Ter) in a child with ID [69].

The *DCAF12L2* gene is located within the locus on Xq25. Large CNVs spanning this region have been reported in patients with developmental delay, intellectual disability, and ASD [70]. The locus on Xq26.3 harbors many genes, but the strongest association signal was located within *ARHGEF6*. This region is linked to X-linked mental retardation [62]. Moreover, mutations in *ARHGEF6* have been reported in patients with X-linked mental retardation [47] and in multiple families with ASD [11,12,71,72].

Although the association signals did not reach genome-wide significance (*p* < 5 × 10^−8^) in our study, many were located within genes that have been implicated in ASD or related neurodevelopmental disorders.

Moreover, three of the top associated SNPs were significantly associated with gene expression suggesting a regulatory function. We also found evidence of association signals in two previously reported, genome-wide significant, ASD susceptibility loci (rs10099100 and rs4299400) suggesting common genetic architecture across populations for these two loci. However, we found no evidence of replication in our dataset for SNPs previously associated with ASD at *p* < 1 × 10^−5^ from the GWAS catalog. This is consistent with a previous meta-analysis of 14 ASD cohorts (totaling 7387 ASD subject and 8567 control) [24], which did not identify any genome-wide significant locus, and few signals were consistent among the 14 different studies. We found that European-derived PRS were not significantly associated with ASD in our cohort. Despite being consistent with previous reports that showed PRS derived from European populations have lower predictive performance when applied to middle eastern populations [37], proper evaluation of PRS requires further studies with larger sample sizes and higher number of SNPs. In our study, we were only able to evaluate 24 out of the 466 SNPs reported by Grove et al. [23].

A limitation of this study is the small sample size. However, the family-based approach utilized in this study is less likely to be influenced by population stratifications compared to population-based case-control designs. Another limitation of this study is the lack of genotype imputation, but this was not performed due to the absence of appropriate reference haplotype panels for the Middle Eastern population to allow accurate imputation of genotypes. Our data warrant further functional studies and replication to confirm their association with ASD and provide further insights into the genetic architecture of ASD.

## 5. Conclusions

This study has identified suggestive evidence of association between ASD and common SNPs in several genes and loci that are suspected of playing a role in ASD or related neurodevelopmental disorders. Our results are consistent with previous studies which showed that common genetic variations in multiple loci contribute to ASD-susceptibility and that the genetic architecture of ASD is complex and shared with other neurodevelopmental disorders. This study has provided new insights into the genetic architecture of ASD in the Middle East.

## Figures and Tables

**Table 1:** Summary of SNPs suggestively associated with ASD.

SNP	Chr	Position	A1	A2	A1 Freq (%)	*p*	OR	95% CI	Genes
rs16823191	2	171,930,818	G	A	6.0	3.8 × 10^−6^	1.22	1.09–1.36	*TLK1, GORASP2*
rs13103662	4	157,249,803	A	G	18.0	5.0 × 10^−6^	0.89	0.83–0.95	*---*
rs2295926	9	101,593,825	G	A	29.5	2.6 × 10^−6^	0.90	0.84–0.95	*GABBR2, ANKS6, GALNT12*
rs11072298	15	71,854,982	A	C	18.2	6.6 × 10^−6^	0.89	0.83–0.95	*THSD4*
rs2368671	X	71,523,650	T	C	8.1	6.4 × 10^−6^	1.15	1.06–1.24	*PIN4, ERCC6L, RPS4X, CITED1, HDAC8*
rs2186039	X	125,384,433	C	A	41.2	2.5 × 10^−6^	1.08	1.04–1.13	*DCAF12L2*
rs12557857	X	135,868,083	G	T	2.2	3.5 × 10^−6^	1.29	1.12–1.48	*ARHGEF6*

Chr, chromosome; Freq, frequency; OR, odds ratio for allele A1; CI, confidence interval. Positions are in reference to human genome build GRCh37.

## Data Availability

All data supporting the findings of this study are available either within the article, the supplementary data, or from the authors upon reasonable request.

## Supplementary Materials

The following are available online at https://www.mdpi.com/article/10.3390/genes12050761/s1, Supplementary Table S1: Family structure of the study subjects, Supplementary Table S2: Replication signals for the lead SNP rs10099100, Supplementary Table S3: Replication analysis for SNPs with *p* < 1 × 10^−5^ from the GWAS catalog, Supplementary Figure S1: Regional association plot for chr 2q31.1 (a) and 4q32.1 (b) regions, Supplementary Figure S2: Regional association plot for chr 9q22.33 (a) and 15q23 (b) regions, Supplementary Figure S3: Regional association plot for chr Xq13.1 (a) and Xq25 (b) regions, Supplementary Figure S4: Regional association plot for chromosome Xq26.3 region, Supplementary Figure S5: *PIN4* expression in multiple tissues in relation to rs2368671 genotype, Supplementary Figure S6: *DCAF12L2* expression in multiple tissues in relation to rs2186039 genotype, Supplementary Figure S7: *ARHGEF6* expression in multiple tissues in relation to rs12557857 genotype.
