# Supplementary material for: Family-Based Genome-Wide Association Study of Autism Spectrum Disorder in Middle Eastern Families
Source: Genes (Basel). 2021 May 18;12(5):761. doi: 10.3390/genes12050761 (PMC8157263; doi:10.3390/genes12050761)
Supplement: Supplementary file 1 [file genes-12-00761-s001.zip › genes-1173379-SI.pdf]

Family Based Genome-Wide Association Study of Autism Spectrum Disorder in Middle Eastern Families

Yasser Al-Sarraj[1,2], Eman Al-Dous[1,2], Rowaida Z. Taha[2], Dina Ahram[2,3], Fouad Alshaban[2], Mohammed Tolfat[4], Hatem El-Shanti[2,5] and Omar M.E. Albagha[1,2,6*]

[1] College of Health and Life Sciences, Hamad Bin Khalifa University, Doha 34110, Qatar; yalsarraj@qf.org.qa (Y.A.-S.); eymaldous@hbku.edu.qa (E.A.-D.)
[2] Qatar Biomedical Research Institute (QBRI), Hamad Bin Khalifa University, Doha 34110, Qatar; rtaha@hbku.edu.qa (R.Z.T.); da2747@cumc.columbia.edu (D.A.); falshaban@hbku.edu.qa (F.A.); hatem-el-shanti@uiowa.edu (H.E.-S.)
[3] Division of Nephrology, Columbia University Medical Center, New York, NY 10032, USA
[4] The Shafallah Center for Children with Special Needs, Doha 33123, Qatar; Mohamed.Tolefat@shafallah.org.qa
[5] Department of Pediatrics, Carver College of Medicine, University of Iowa, Iowa City, IA 52242, USA
[6] Centre for Genomic and Experimental Medicine, Institute of Genetics and Molecular Medicine, University of Edinburgh, Edinburgh EH4 2XU, UK
* Correspondence: oalbagha@hbku.edu.qa

Supplementary Materials

Supplementary Table S1. Family structure of the study subjects

Number of Families	Unaffected parents	Child with ASD	Unaffected Child	Total number of subjects
103	2	1	0	309
1	2	2	1	5
22	2	0	1	66
28	1	1	0	56
6	1	0	1	12
1	2	1	1	4
1	2	0	2	4
1	1	2	0	3
1	1	3	0	4
7	0	1	0	7
4	2	0	0	8
9	1	0	0	9

Supplementary Table S2. Replication signals for the lead SNP rs10099100*.

		iPSYCH Data		LD from EUR 1000 Genome		Our Data	
Proxy SNP	Bp to lead SNP	P-value	OR	D'	R2	P-value	OR
rs6601521	-52172	0.001	0.95	0.93	0.30	0.013	0.95
rs7826189	-51085	0.024	1.03	0.53	0.14	0.04	1.04
rs733396	-45206	0.01	1.03	0.53	0.14	0.003	1.06
rs6601518	-28459	0.01	1.03	0.58	0.21	0.038	1.04
rs7827623	35844	0.007	0.95	0.90	0.37	0.03	0.95

*We searched for close proxies (within 100 kb) in Linkage disequilibrium (LD) with the lead SNP based on LD data from European population form 1000 Genome (since the previously identified lead SNPs were found in studies of European populations). We then checked if the proxies were genotyped in our data set and compared results.

Supplementary Table S3. Replication analysis for SNPs with P<1x10^{-5} from the GWAS catalog*.

	GWAS Catalog			Our Data			Effect	
SNP	Effect allele	OR	P	Effect Allele	OR	P	Same Direction	PMID
rs910805	NA	NA	1.00E-10	G	1.00	0.88	NA	32606422
rs8453	G	NA	3.00E-08	G	0.92	0.08	NA	24189344
rs6508210	NA	NA	3.00E-08	C	1.00	0.95	NA	32606422
rs6537825	A	1.4	3.00E-08	A	0.98	0.58	No	24189344
rs3827735	C	NA	6.00E-08	C	1.04	0.13	NA	24189344
rs6537825	A	NA	6.00E-08	A	0.98	0.58	NA	24189344
rs11585926	T	NA	6.00E-08	T	0.97	0.19	NA	24189344
rs11587400	C	NA	7.00E-08	C	1.00	0.93	NA	24189344
rs10858047	T	NA	8.00E-08	T	1.02	0.64	NA	24189344
rs10489525	G	NA	9.00E-08	G	1.02	0.47	NA	24189344
rs8453	G	NA	9.00E-08	G	0.92	0.08	NA	24189344
rs4150167	NA	1.96	3.00E-07	A	1.03	0.43	NA	22843504
rs910805	G	1.08	3.00E-07	G	1.00	0.88	Yes	30804558
rs2391769	G	1.07	5.00E-07	G	1.01	0.59	Yes	30804558
rs2635182	T	1.07	5.00E-07	T	1.02	0.31	Yes	30804558
rs1452075	T	1.07	6.00E-07	T	0.98	0.49	No	30804558
rs7711337	NA	1.22	8.00E-07	A	1.04	0.07	NA	22843504
rs7834018	NA	1.56	8.00E-07	G	1.00	0.98	NA	22843504
rs11185408	G	1.06	1.00E-06	G	0.98	0.29	No	30804558
rs6538761	A	1.24	2.00E-06	A	1.00	0.82	Yes	24189344
rs10038113	T	1.33	3.00E-06	T	0.97	0.21	No	19456320
rs4916723	C	1.06	3.00E-06	C	1.00	0.93	Yes	30804558
rs7142002	NA	1.56	3.00E-06	G	1.05	0.16	NA	20663923
rs10742763	T	1.31	4.00E-06	T	1.14	0.06	Yes	32747698
rs6453278	NA	1.25	5.00E-06	C	1..01	0.82	NA	19812673
rs7627815	T	1.38	8.00E-06	T	0.95	0.36	No	32747698
rs2280840	G	NA	9.00E-06	G	1.00	0.99	NA	32996047
rs6599528	A	NA	9.00E-06	A	1.00	0.83	NA	32996047
rs2447097	A	1.53	9.00E-06	A	1.00	0.82	Yes	26398136

*GWAS catalog data were retrieved from https://www.ebi.ac.uk/gwas/home using autism or autism spectrum disorder as phenotype. OR; odds ratio for effect allele, PMID; PubMed ID.

Supplementary Figure S1. Regional association plot for chr 2q31.1 (**a**) and 4q32.1 (**b**) regions

a

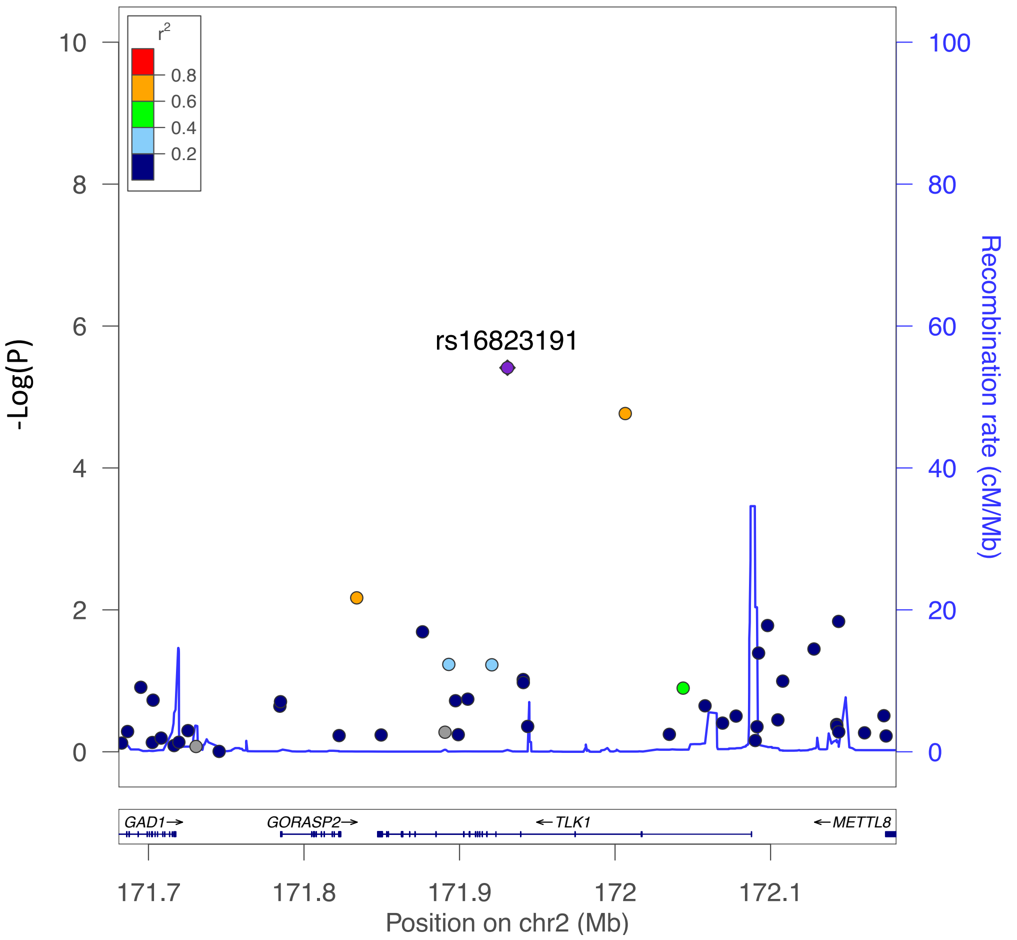

b

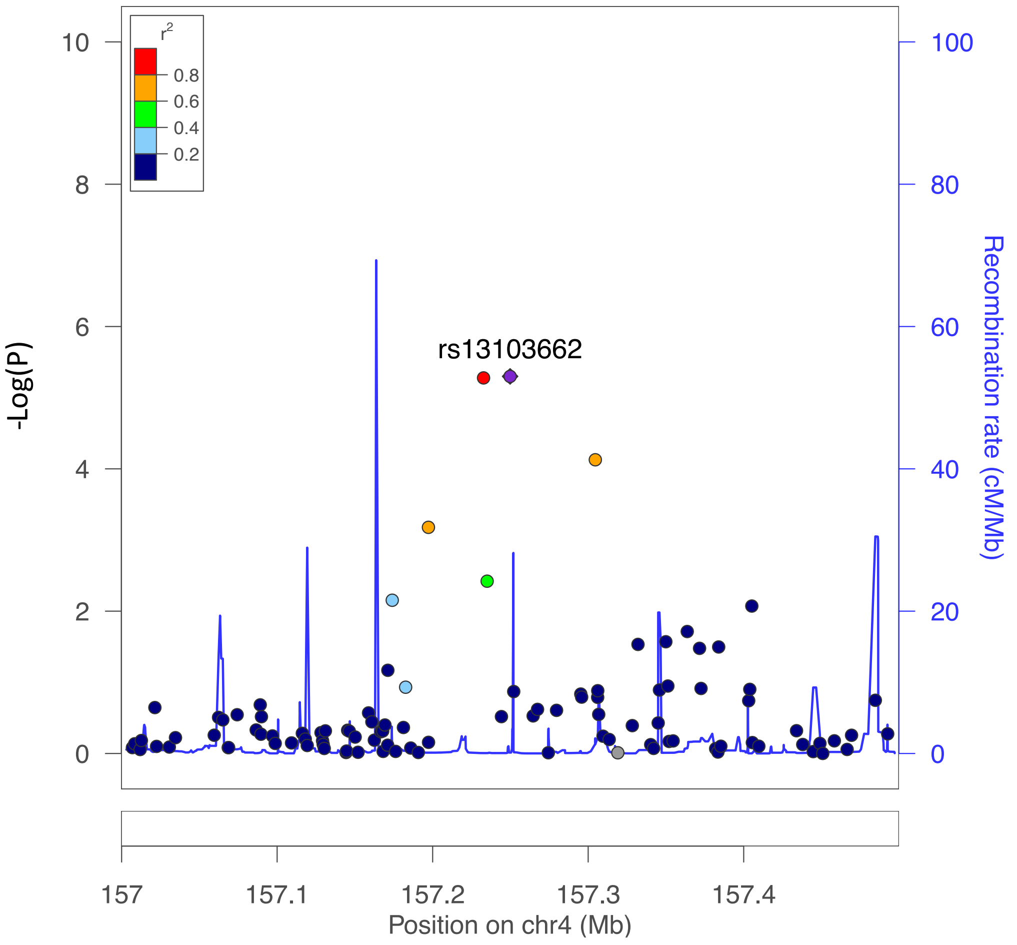

Supplementary Figure S2. Regional association plot for chr 9q22.33 (**a**) and 15q23 (**b**) regions

a

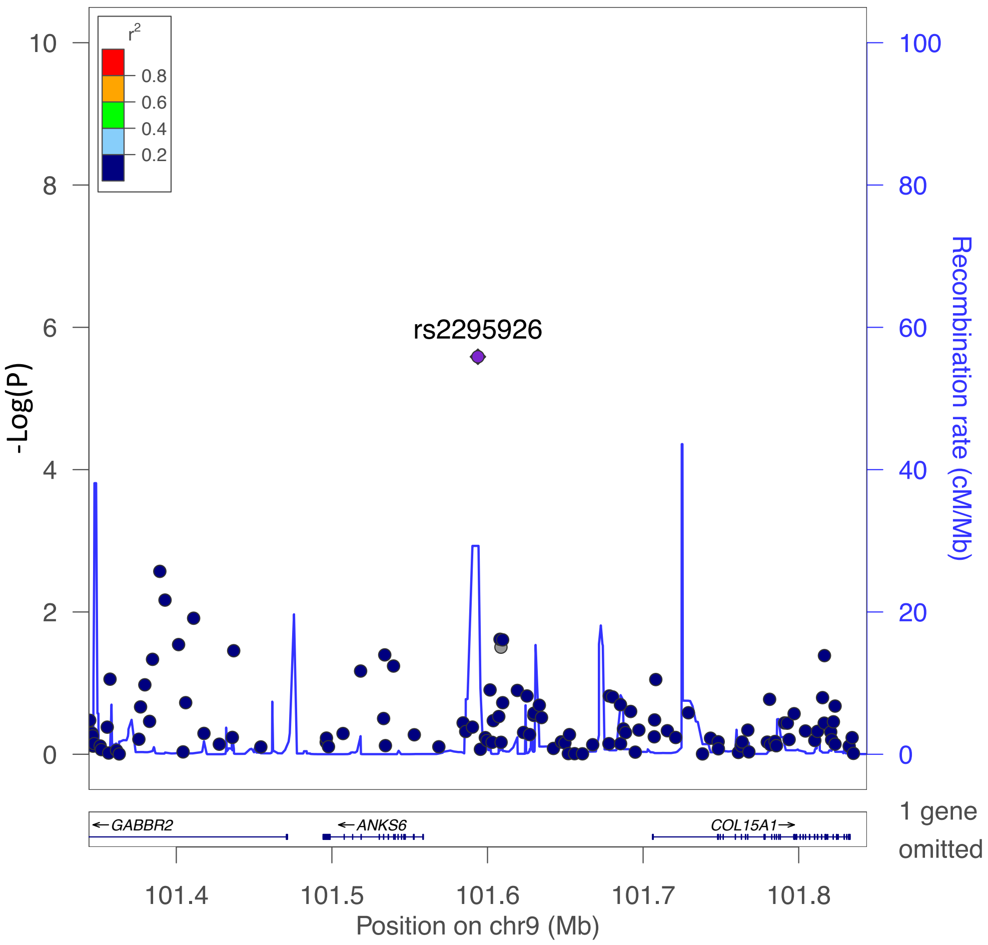

b

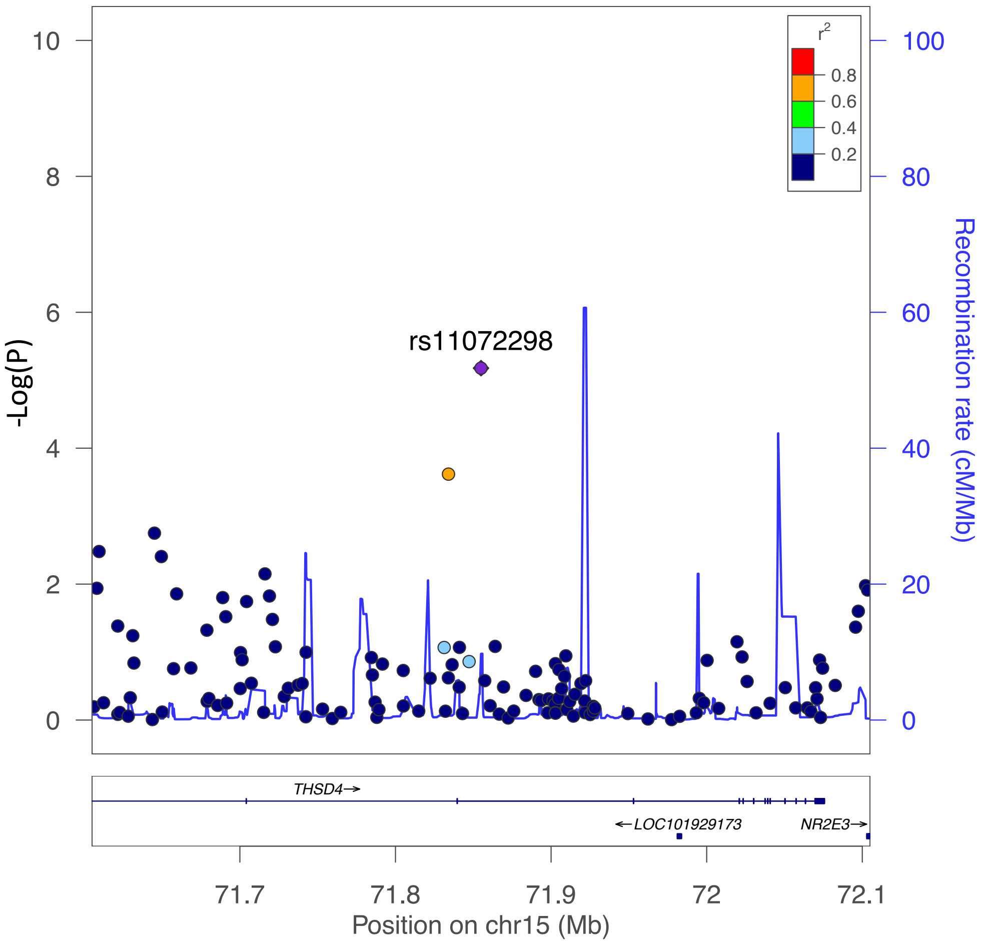

Supplementary Figure S3. Regional association plot for chr Xq13.1 (**a**) and Xq25 (**b**) regions

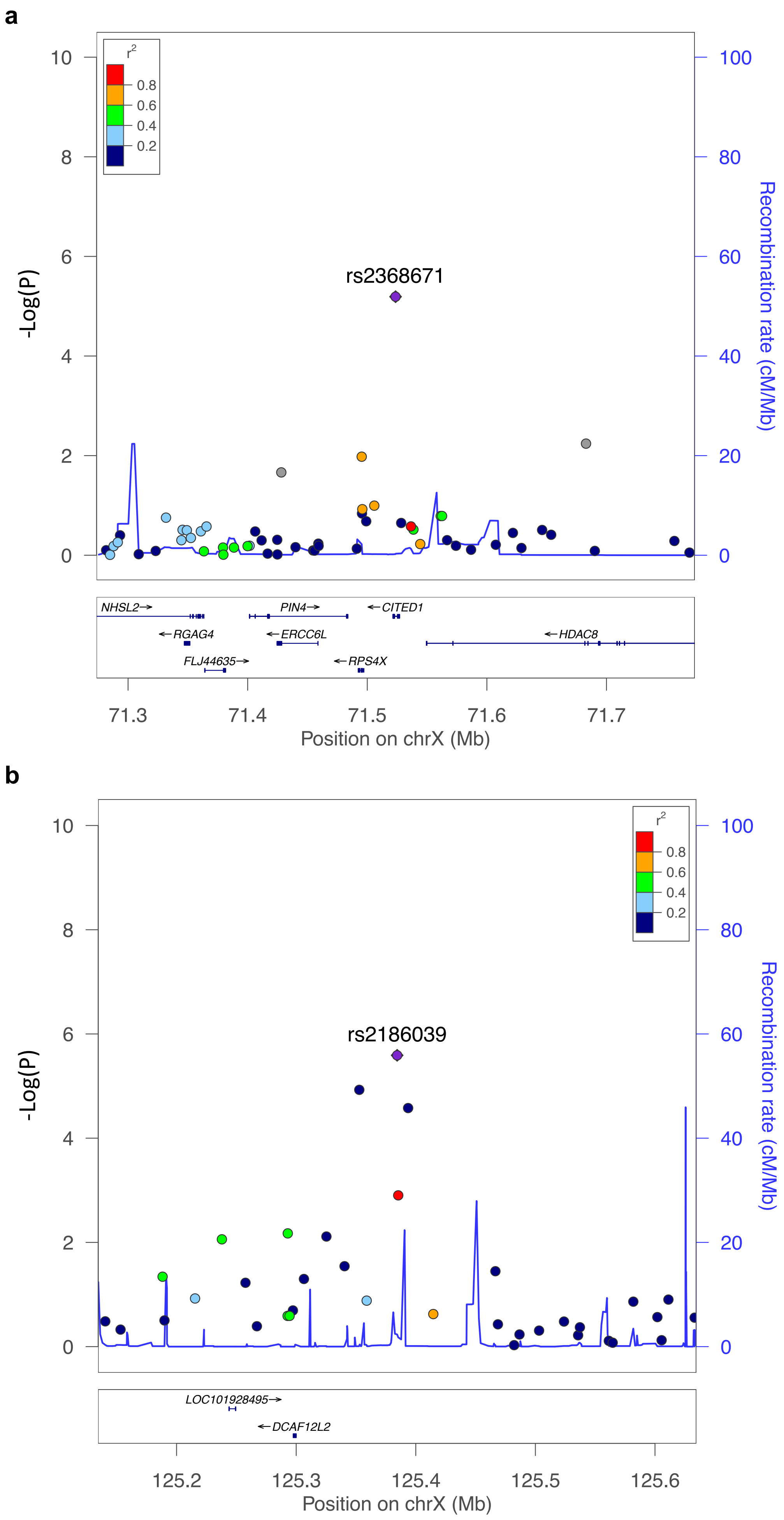

Supplementary Figure S4. Regional association plot for chromosome Xq26.3 region.

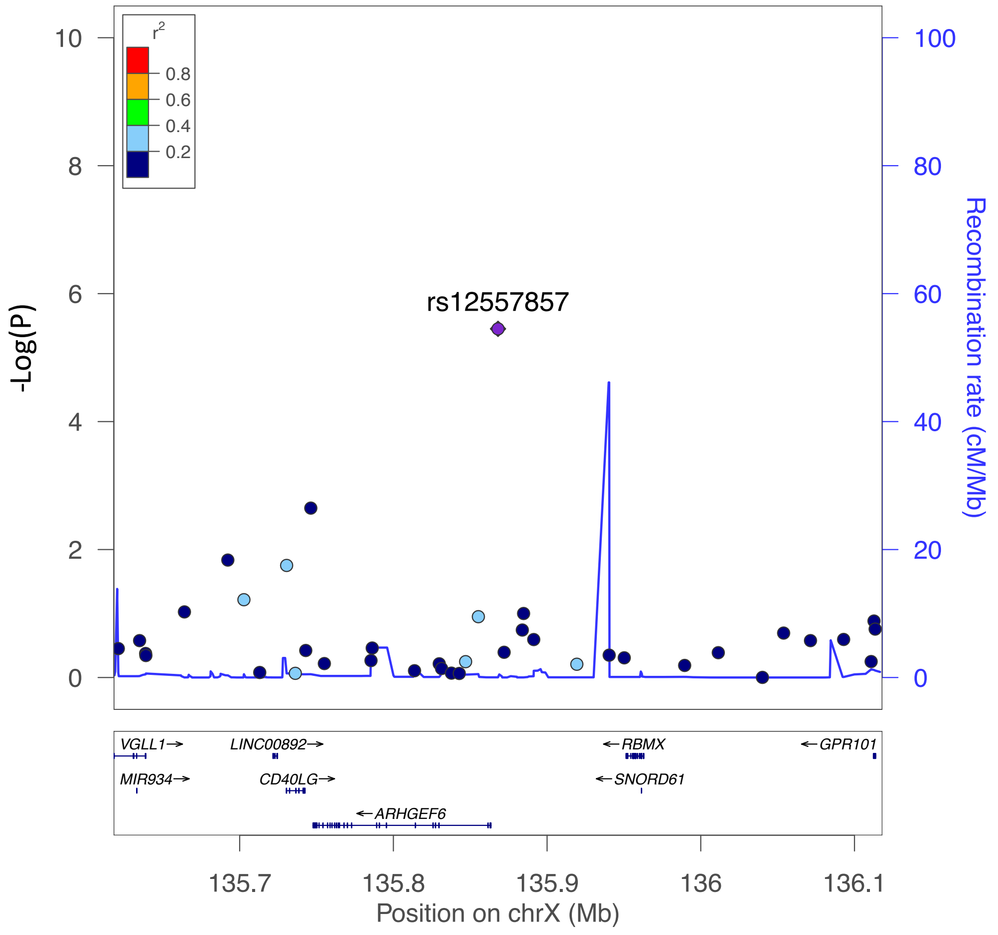

Supplementary Figure S5. *PIN4* expression in multiple tissues in relation to rs2368671 genotype.

Tissue	Samples	NES	p-value	m-value
Uterus	129	0.444	0.03	1.00
Esophagus - Mucosa	497	0.406	1.5e-12	1.00
Liver	208	0.391	2.0e-6	1.00
Cells - EBV-transformed lymphocytes	147	0.373	2.6e-4	1.00
Artery - Aorta	387	0.301	3.7e-6	1.00
Breast - Mammary Tissue	396	0.300	5.8e-8	1.00
Artery - Coronary	213	0.297	9.1e-4	1.00
Esophagus - Muscularis	465	0.296	1.2e-7	1.00
Esophagus - Gastroesophageal Junction	330	0.291	1.8e-4	1.00
Small Intestine - Terminal Ileum	174	0.289	5.4e-4	1.00
Colon - Sigmoid	318	0.288	4.1e-6	1.00
Vagina	141	0.285	0.06	0.994
Spleen	227	0.280	8.9e-4	1.00
Stomach	324	0.278	1.6e-5	1.00
Skin - Sun Exposed (Lower leg)	605	0.264	3.2e-9	1.00
Skin - Not Sun Exposed (Suprapubic)	517	0.261	3.6e-8	1.00
Ovary	167	0.254	0.07	0.977
Colon - Transverse	368	0.247	1.2e-5	1.00
Pituitary	237	0.232	1.2e-3	1.00
Artery - Tibial	584	0.231	6.6e-9	1.00
Prostate	221	0.213	1.5e-3	1.00
Pancreas	305	0.205	0.008	0.998
Minor Salivary Gland	144	0.201	0.06	0.961
Adipose - Visceral (Omentum)	469	0.197	2.8e-5	1.00
Whole Blood	670	0.194	9.8e-7	1.00
Adipose - Subcutaneous	581	0.188	8.8e-7	1.00
Heart - Left Ventricle	386	0.187	9.7e-5	1.00
Adrenal Gland	233	0.186	0.008	1.00
Thyroid	574	0.181	1.4e-5	1.00
Lung	515	0.179	3.0e-6	1.00
Cells - Cultured fibroblasts	483	0.167	1.6e-6	1.00
Muscle - Skeletal	706	0.157	1.3e-6	1.00
Heart - Atrial Appendage	372	0.150	0.008	0.999
Brain - Hippocampus	165	0.145	0.01	0.986
Testis	322	0.133	2.9e-4	0.970
Brain - Caudate (basal ganglia)	194	0.131	0.03	0.977
Nerve - Tibial	532	0.126	3.2e-3	0.983
Brain - Hypothalamus	170	0.123	0.1	0.927
Brain - Frontal Cortex (BA9)	175	0.120	0.08	0.980
Kidney - Cortex	73	0.116	0.2	0.928
Brain - Substantia nigra	114	0.0891	0.5	0.915
Brain - Amygdala	129	0.0783	0.3	0.786
Brain - Spinal cord (cervical c-1)	126	0.0506	0.6	0.669
Brain - Putamen (basal ganglia)	170	0.0462	0.6	0.705
Brain - Cerebellar Hemisphere	175	0.0426	0.5	0.389
Brain - Anterior cingulate cortex (BA24)	147	0.0390	0.6	0.609
Brain - Cerebellum	209	0.0374	0.6	0.480
Brain - Cortex	205	0.0221	0.8	0.428
Brain - Nucleus accumbens (basal ganglia)	202	-0.00813	0.9	0.0850

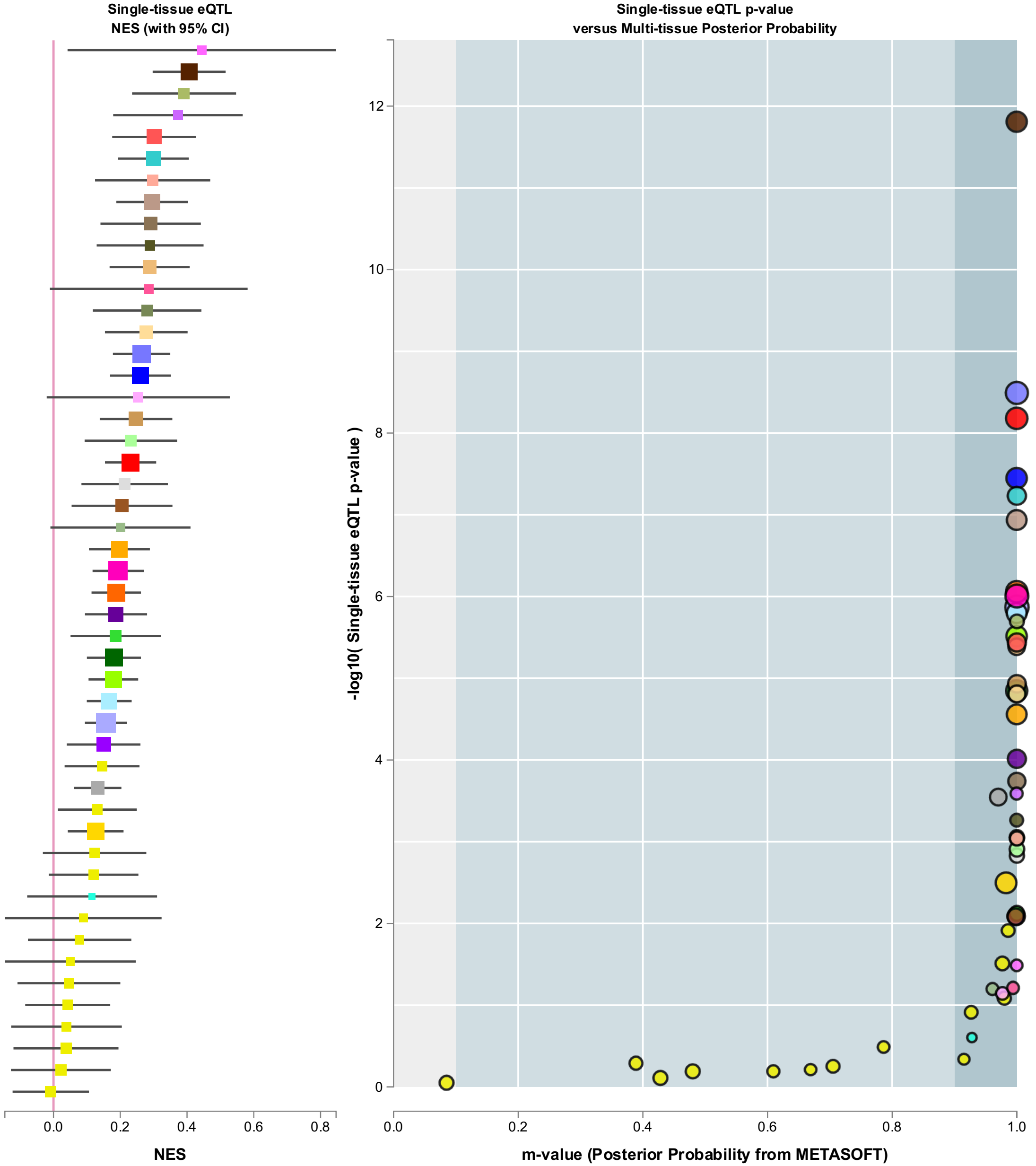

Adapted from GTEX portal ([https://gtexportal.org/home/).](https://gtexportal.org/home/)

Supplementary Figure S6. *DCAF12L2* expression in multiple tissues in relation to rs2186039 genotype

Tissue	Samples	NES	p-value	m-value
Brain - Hippocampus	165	0.0611	0.2	0.0310
Brain - Amygdala	129	0.0518	0.5	0.437
Brain - Frontal Cortex (BA9)	175	0.0199	0.7	0.0490
Testis	322	0.0150	0.4	0.00
Cells - EBV-transformed lymphocytes	147	-	-	-
Heart - Left Ventricle	386	-	-	-
Liver	208	-	-	-
Lung	515	-	-	-
Pancreas	305	-	-	-
Spleen	227	-	-	-
Whole Blood	670	-	-	-
Brain - Anterior cingulate cortex (BA24)	147	-0.0249	0.6	0.393
Brain - Caudate (basal ganglia)	194	-0.0331	0.4	0.352
Brain - Spinal cord (cervical c-1)	126	-0.0377	0.6	0.648
Brain - Cortex	205	-0.0384	0.4	0.508
Muscle - Skeletal	706	-0.0461	0.2	0.437
Prostate	221	-0.0469	0.4	0.717
Brain - Substantia nigra	114	-0.0480	0.3	0.614
Brain - Putamen (basal ganglia)	170	-0.0508	0.2	0.686
Colon - Transverse	368	-0.0527	0.07	0.590
Brain - Hypothalamus	170	-0.0742	0.03	0.953
Skin - Sun Exposed (Lower leg)	605	-0.0794	0.009	0.941
Cells - Cultured fibroblasts	483	-0.0795	0.1	0.889
Minor Salivary Gland	144	-0.0907	0.2	0.815
Adrenal Gland	233	-0.0930	0.06	0.913
Brain - Nucleus accumbens (basal ganglia)	202	-0.0952	2.4e-3	0.992
Breast - Mammary Tissue	396	-0.0974	0.03	0.976
Small Intestine - Terminal Ileum	174	-0.102	0.05	0.934
Skin - Not Sun Exposed (Suprapubic)	517	-0.103	0.007	0.996
Thyroid	574	-0.113	2.5e-3	1.00
Stomach	324	-0.121	0.01	0.987
Vagina	141	-0.126	0.1	0.858
Pituitary	237	-0.132	0.008	0.976
Adipose - Subcutaneous	581	-0.133	8.3e-8	1.00
Heart - Atrial Appendage	372	-0.135	0.02	0.982
Artery - Tibial	584	-0.141	2.6e-7	1.00
Adipose - Visceral (Omentum)	469	-0.143	6.6e-4	1.00
Uterus	129	-0.147	0.1	0.896
Esophagus - Gastroesophageal Junction	330	-0.154	6.1e-5	1.00
Nerve - Tibial	532	-0.162	2.1e-6	1.00
Esophagus - Muscularis	465	-0.167	1.7e-6	1.00
Artery - Coronary	213	-0.180	3.8e-3	0.997
Kidney - Cortex	73	-0.181	0.3	0.840
Brain - Cerebellar Hemisphere	175	-0.189	0.006	0.964
Colon - Sigmoid	318	-0.208	3.7e-8	1.00
Brain - Cerebellum	209	-0.208	4.3e-3	1.00
Esophagus - Mucosa	497	-0.210	6.7e-6	1.00
Artery - Aorta	387	-0.236	1.2e-5	1.00
Ovary	167	-0.243	0.006	0.981

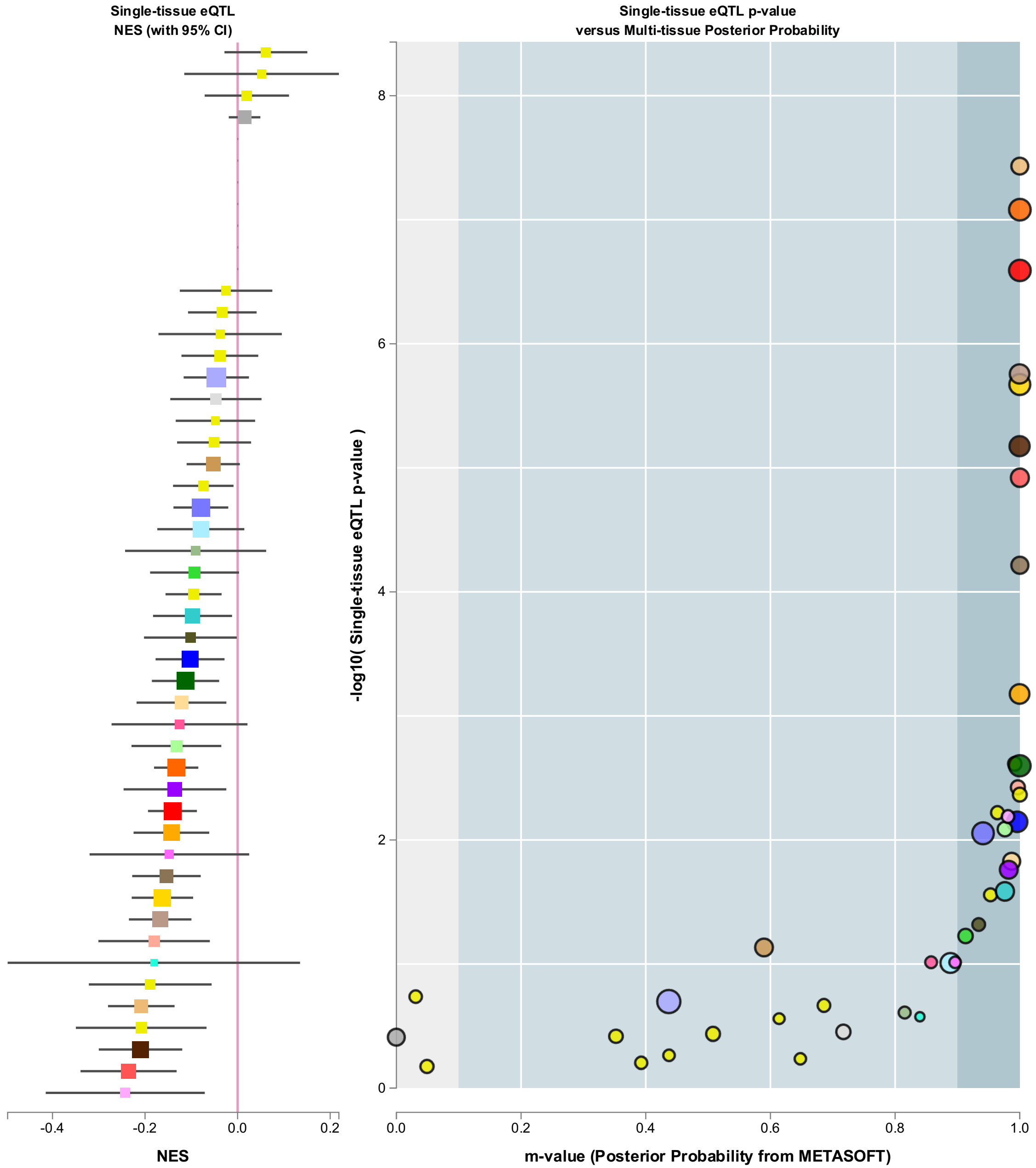

Adapted from GTEX portal (https://gtexportal.org/home/).

Supplementary Figure S7. *ARHGEF6* expression in multiple tissues in relation to rs12557857 genotype

Tissue	Samples	NES	p-value	m-value
Heart - Atrial Appendage	372	0.304	1.5e-4	0.987
Ovary	167	0.239	0.09	0.761
Pancreas	305	0.221	0.01	0.868
Brain - Anterior cingulate cortex (BA24)	147	0.209	0.03	0.841
Artery - Aorta	387	0.198	9.2e-4	0.977
Brain - Cerebellum	209	0.187	2.7e-3	0.979
Brain - Frontal Cortex (BA9)	175	0.158	0.04	0.892
Heart - Left Ventricle	386	0.155	2.2e-4	0.999
Brain - Caudate (basal ganglia)	194	0.154	0.02	0.904
Brain - Cortex	205	0.150	0.009	0.951
Minor Salivary Gland	144	0.122	0.2	0.783
Esophagus - Muscularis	465	0.115	0.01	0.964
Brain - Cerebellar Hemisphere	175	0.114	0.1	0.825
Artery - Tibial	584	0.111	0.007	0.966
Thyroid	574	0.0995	2.0e-3	0.985
Muscle - Skeletal	706	0.0936	0.01	0.988
Brain - Spinal cord (cervical c-1)	126	0.0851	0.3	0.700
Liver	208	0.0816	0.4	0.623
Brain - Substantia nigra	114	0.0807	0.3	0.677
Testis	322	0.0754	0.1	0.791
Prostate	221	0.0604	0.2	0.655
Cells - Cultured fibroblasts	483	0.0592	0.1	0.645
Nerve - Tibial	532	0.0553	0.1	0.664
Spleen	227	0.0532	0.5	0.533
Brain - Amygdala	129	0.0464	0.6	0.533
Brain - Putamen (basal ganglia)	170	0.0421	0.6	0.554
Skin - Not Sun Exposed (Suprapubic)	517	0.0410	0.3	0.470
Stomach	324	0.0390	0.5	0.475
Artery - Coronary	213	0.0372	0.7	0.534
Esophagus - Mucosa	497	0.0340	0.4	0.347
Brain - Nucleus accumbens (basal ganglia)	202	0.0337	0.6	0.492
Brain - Hypothalamus	170	0.0323	0.6	0.416
Lung	515	0.0318	0.4	0.225
Brain - Hippocampus	165	0.0244	0.8	0.475
Adipose - Visceral (Omentum)	469	0.0167	0.7	0.332
Esophagus - Gastroesophageal Junction	330	0.0118	0.8	0.252
Colon - Transverse	368	0.00864	0.8	0.0490
Adrenal Gland	233	0.00272	1	0.439
Small Intestine - Terminal Ileum	174	0.00155	1	0.305
Colon - Sigmoid	318	-0.00295	0.9	0.174
Whole Blood	670	-0.00572	0.8	0.0140
Skin - Sun Exposed (Lower leg)	605	-0.00693	0.8	0.0520
Cells - EBV-transformed lymphocytes	147	-0.0229	0.9	0.461
Pituitary	237	-0.0309	0.6	0.220
Breast - Mammary Tissue	396	-0.0617	0.3	0.106
Kidney - Cortex	73	-0.0657	0.6	0.438
Vagina	141	-0.0684	0.5	0.294
Adipose - Subcutaneous	581	-0.0711	0.2	0.0150
Uterus	129	-0.136	0.5	0.397

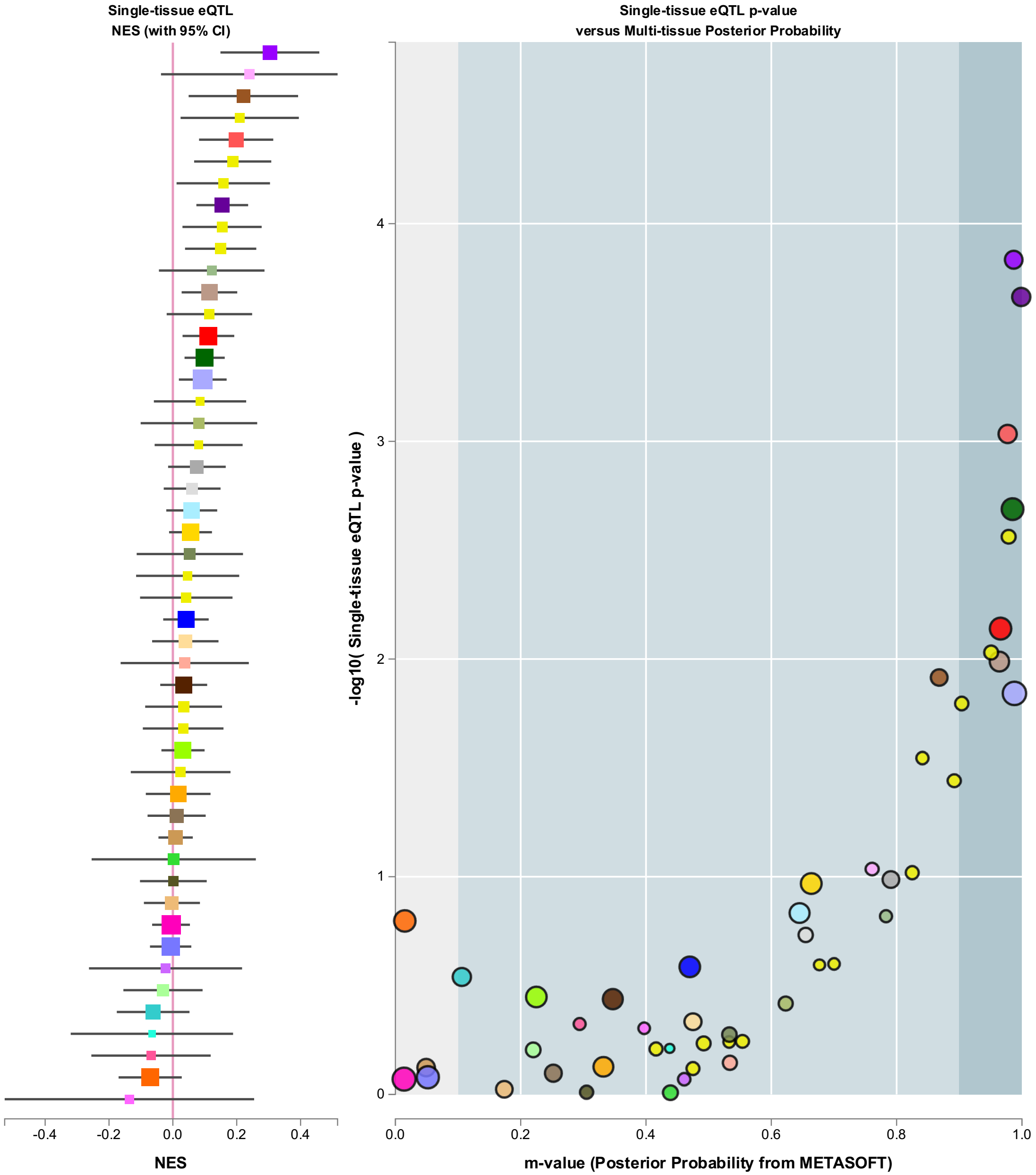

Adapted from GTEX portal (https://gtexportal.org/home/).
